# Development and validation of a sensitive LC-MS/MS method for pioglitazone: application towards pharmacokinetic and tissue distribution study in rats†

**DOI:** 10.1039/d1ra01126j

**Published:** 2021-03-18

**Authors:** Kusuma Kumari G., Praveen Thaggikuppe Krishnamurthy, Ravi Kiran Ammu V. V. V., Kurawattimath Vishwanath, S. T. Narenderan, B. Babu, Nagappan Krishnaveni

**Affiliations:** Department of Pharmacology, JSS College of Pharmacy, JSS Academy of Higher Education & Research Ooty-643 001 The Nilgiris Tamil Nadu India praveentk7812@gmail.com praveentk@jssuni.edu.in; Drug Metabolism and Pharmacokinetics-Toxicology Division, Sai Advantium Pharma Ltd. Pune 411 057 India; Department of Pharmaceutical Analysis, JSS College of Pharmacy, JSS Academy of Higher Education & Research Ooty-643 001 The Nilgiris Tamil Nadu India

## Abstract

In the present study, a sensitive LC-MS/MS method was developed and validated to measure pioglitazone (PGZ) concentrations in rat plasma and tissues. The chromatographic separation was achieved by using a YMC Pro C_18_ column (100 mm × 4.6 mm, 3μ) with a mobile phase consisting of formic acid (0.1% v/v) and acetonitrile (5 : 95) at a flow rate of 0.7 mL min^−1^ and injection volume of 10 μL (IS: rosiglitazone). Mass spectrometric detection was done using triple quadrupole mass spectrometry using the ESI interface operating in a positive ionization mode. The developed method was validated over a linearity range of 1–500 ng mL^−1^ with detection and a lower quantification limit of 0.5 ng mL^−1^ and 1 ng mL^−1^. The method accuracy ranged from 95.89–98.78% (inter-day) & 93.39–97.68% (intra-day) with a precision range of 6.09–8.12% for inter-day & 7.55–9.87% for intra-day, respectively. The PGZ shows the highest *C*_max_ of 495.03 ng mL^−1^ in plasma and the lowest *C*_max_, 24.50 ± 2.71 ng mL^−1^ in bone. The maximum *T*_max_ of 5.00 ± 0.49 h was observed in bone and a minimum of 1.01 ± 0.05 h in plasma. The AUC_(0–24 h and 0–*∞*)_ values are highest in plasma (1056.58 ± 65.78 & 1069.38 ± 77.50 ng h^−1^ mL^−1^) and lowest in brain (166.93 ± 15.70 &167.12 ± 16.77 ng h^−1^ mL^−1^), and the *T*_1/2_ was highest in plasma (5.62 ± 0.74 h) and lowest in kidney (2.78 ± 0.19). The developed method was successfully used to measure the PGZ pharmacokinetic and tissue distribution. Further, the developed method could be utilized for validating target organ (adipose tissue) specific delivery of PGZ (nano-formulations) in addition to conventional dosage forms.

## Abstract 2

The developed method was investigated for target and off-target distribution of pioglitazone and could be applied to validate the site-specific delivery systems.

## Graphical abstract

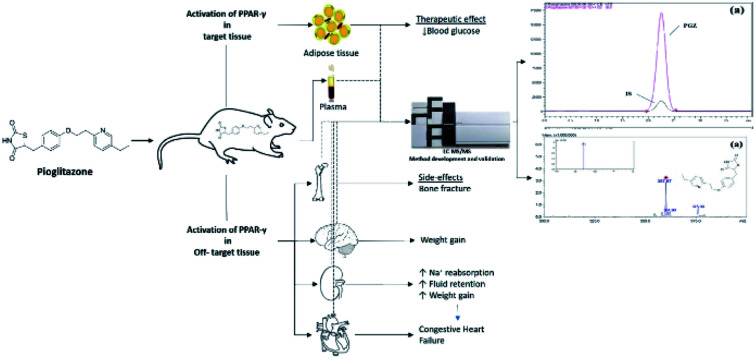

## 1 Introduction

Glitazones or thiazolidinediones are a class of insulin-sensitizers used in the treatment of type-2 diabetes mellitus (T2DM).^1^ They improve insulin sensitivity (without promoting insulin release from pancreatic β-cells) and increase peripheral glucose utilization and reduce hepatic glucose production, and as a result, they reduce both preload and afterload on β-cells.^2^ Unlike sulfonylureas, these agents do not associate with hypoglycaemic side effects.^1,3^ The above advantages, therefore, make them as a preferred agent for the treatment of T2DM. Pioglitazone (PGZ) and rosiglitazone (RGZ) were approved in the year 1999 by USFDA for the management of T2DM.^4,5^ Although these drugs were found to be safe on the hepatic system, later some serious side effects associated with RGZ (weight gain; fluid retention, bone loss, and congestive heart failure) and PGZ (bladder cancer, weight gain), were reported, resulting in WHO restriction of RGZ and a label change for PGZ for risk of bladder cancer.^4,6–11^ The above off-target side effects associated with RGZ and PGZ are reported to be due to the activation of PPAR-γ (target receptor) in the non-adipose tissues such as kidney, bone, brain, and heart.^11–17^ To overcome RGZ and PGZ, off-target side effects, researches are employing nano-drug delivery systems (such as solid lipid nanoparticles, dendrimers, carbon nanomaterials, metallic nanoparticles, *etc.*) to achieve target-specific delivery of these drugs to adipose tissue.^18^ In this context, it becomes apparent to develop sensitive analytical methods to measure the concentration of glitazones in different matrices including, blood, adipose, brain, bone, heart, *etc.* to demonstrate the site-specific delivery of novel drug delivery systems.^15,19–22^ Further, the reported methods have their individual advantages such as short run time, simple sample treatment technique, requiring low plasma volume and improved method sensitivity.^19,20,23^ However, it is significant to have individual methods with all the advantages. Hence, in the present study, we have developed and validated a sensitive liquid chromatography-tandem mass spectrometry method (LC-MS/MS) for measuring PGZ concentration in rat plasma and tissues (adipose, heart, brain, bone, and kidney). Further, the method can be used for validating site-specific delivery systems of PGZ in addition to conventional dosage forms.

## 2 Experimental

### 2.1 Chemicals and reagents

PGZ (purity 99.98%) and RGZ (purity 99.96%) (internal standard, IS) were obtained in the form of a gift sample from Wallace Pharmaceutical Pvt. Ltd., Goa, India. LC-MS grade formic acid and acetonitrile were procured from Sisco Research Lab Pvt. Ltd., Mumbai, India. Other reagents were of analytical grade from SD Fine Chemicals, Mumbai, India. The ultrapure water was obtained using the Milli-Q RO system, Millipore, India.

### 2.2 Preparation of standard samples for linearity and quality control

For the linearity curve generation, the standard stock solution of PGZ (1 mg mL^−1^) was prepared in acetonitrile/plasma/tissue homogenate (10% w/v). Working stock solution of PGZ (1, 5, 10, 25, 50, 100, 250, and 500 ng mL^−1^) was prepared by diluting the standard stock solution with acetonitrile/plasma/tissue homogenate. For quality control, the PGZ was prepared at a concentration of 450, 100, 15 and 1 ng mL^−1^ in acetonitrile/plasma/tissue homogenate to represent high-quality control (HQC), medium quality control (MQC), low-quality control (LQC), and lower limit quality control (LLQC) samples, respectively. In all these samples, the concentration of IS was 100 ng mL^−1^.

### 2.3 Extraction of the drug from plasma and tissue samples

A simple protein precipitation method was adopted and optimized for the extraction of PGZ from plasma and tissue homogenate. To the plasma (200 μL)/tissue homogenate (10% w/v, 200 μL) taken in 2 mL eppendorf tube a 100 μL of IS (1 μg mL^−1^) was added and the volume was made up to 2 mL with acetonitrile. The tubes were vortexed for 30 seconds, followed by centrifugation (Remi R8C, Mumbai) for 15 min at 5000 rpm. After centrifugation, the upper supernatant acetonitrile layer was separated for LC-MS/MS analysis.

### 2.4 Mass and chromatographic condition

The LC-MS/MS analysis of PGZ was performed using Shimadzu 8030 system with CTO – 20 AC column oven, LC – 20 AD pump, CBM – 20 controllers, SPD – M 20 PDA detector, electrospray ionization (ESI) interface, and SIL – 20 AC autosampler.

**Fig. 1:**
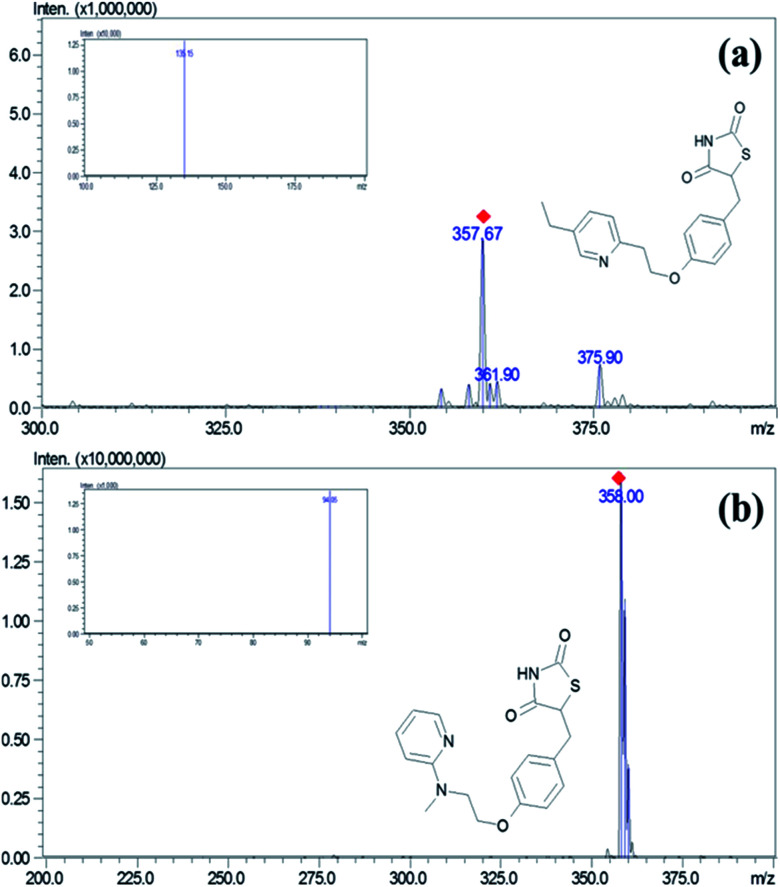
Mass and MRM spectra (image inside the box) of (a) PGZ and (b) IS (RSZ) in positive detection mode.

The separation of the PGZ and IS was performed on a YMC Pro C_18_ column (100 mm × 4.6 mm, 3μ) with a mobile phase consisting of formic acid (0.1% v/v) and acetonitrile (5 : 95, v/v) at a flow rate of 0.7 mL min^−1^ and injection volume of 10 μL. Mass spectrometric detection was done on a triple quadrupole mass spectrometry using the ESI interface operating in a positive ionization mode. The control of the instrument and acquisition of data was carried using Lab solution software (Shimadzu Ltd., Mumbai, India). The block temperature of 350 °C and desolvation temperature of 250 °C was maintained with a detector voltage and CID gas set at 1.3 kV and 230 kPa, respectively. For nebulization and collision, nitrogen (99.95%) and argon gases (99.99%) were used. Multiple reaction monitoring (MRM) mode was used for the quantification of PGZ. MRM transitions of *m*/*z* 357.95 → 135.15 and *m*/*z* → 94.05 with the collision energy of −30 eV and −47 eV were chosen for quantification of PGZ and IS, respectively (Fig. 1).

### 2.5 Method validation

The developed LC-MS/MS method was validated for specificity, linearity, sensitivity, precision, accuracy, matrix effect, % recovery, and stability as per the USFDA bio-analytical method validation guidelines.^24^

### 2.6 Pharmacokinetic and tissue distribution studies in rat

Twelve male Wistar rats (200 ± 20 g) were procured from the in-house animal facility of JSS College of Pharmacy, Ooty. All the rats were housed in standard laboratory conditions (22 ± 2 °C; light/dark cycle of 12 : 12 h; relative humidity 30–70%) with normal pellet diet (M/s Amrit feeds Ltd., Bengaluru, India) and water ad libitum. The study protocol was prior approved by the Institutional Animal Ethics Committee (IAEC) (approval number JSSCP/OT/IAEC/13/2019-20). The overnight fasted animals (8–10 h) were orally administered with PGZ (10 mg kg^−1^), and the blood sample (∼1 mL) was collected from retro-orbital plexus under mild isoflurane anesthesia at 0, 0.5, 1, 3, 5, 7 and 24 h in a time staggered manner as shown in Table SI.† After blood collection, for each time point, one animal was culled using isoflurane anesthesia. The adipose tissue, brain, bone, heart, and kidney were harvested, washed with PBS (0.1 mM, pH-7.4), blotted with tissue paper, and weighed. A 10% w/v tissue homogenate was prepared in PBS (0.1 mM, pH-7.4) and stored at −70 °C until further analysis.^25^ The pharmacokinetic data were analyzed using PK solver software.

## 3 Results

### 3.1 Optimization of LC-MS/MS conditions

The various mobile phase compositions used for LC-MS/MS method development are given in Table SII.† Among the mobile phases employed acetonitrile: formic acid (0.1% v/v) at the ratio of 95 : 5 show a good peak when compared to others. Further, the detection conditions for PGZ and RGZ were optimized by varying the ionization mode (+ve and −ve), dwell time (50–200 ms), Q1 pre-bias (5–30 V), Q3 pre-bias (5–30 V) and collision energy (1–50 eV). The optimized LC-MS/MS conditions employed for the detection of PGZ and RGZ (IS) were given in Table 1.

**Table 1:** The optimized LC-MS/MS detection conditions for PGZ and RGZ (IS)

Analyte	Molecular weight (g mol^−1^)	Ion mode	Product ion	Precursor ion (*m*/*z*)	Dwell time (m s^−1^)	Q1 pre bias (V)	Q3 pre bias (V)	Collision energy (eV)	Retention time (min)
PGZ	356.44	Positive	135.15	357.95	100	−28	−28	−30	2.45
RGZ (IS)	357.42	Positive	94.05	358.00	100	−28	−20	−47	2.46

### 3.2 Method validation

The method validation results are given in Tables 2–4. The developed method shows a linearity range of 1–500 ng mL^−1^ in both plasma and tissue samples with a correlation coefficient (*R*^2^) ranging from 0.992–0.999. Further, the % CV, LOD, and LLOQ for this method in all the biological samples was found to be <2%, 0.5, and 1.0 ng mL^−1^, respectively (Table 2). The results of percentage recovery, absolute matrix effect, intra-, and inter-day accuracy and precision determined at four QC levels (1, 15, 90, 450 ng mL^−1^) in six replicates were found to be within limits (Table 3), indicating that the present method was accurate and reproducible. Further, the stability test results of PGZ carried out in plasma and tissue homogenate under different storage conditions at four QC levels (1, 15, 90, 450 ng mL^−1^) in six replicates show that the PGZ is stable after 3-freeze–thaw cycles at −70 ± 2 °C (% RSD −5.04 to 7.25%) and at 25 °C for 24 h in both tissue matrix (% RSD −7.35 to 11.94%) and stock solution (% RSD −1.47 to 6.91%) (Table 4). The obtained results were found to be within the permissible limits (±20% RSD), confirming that the PGZ was stable in all three testing conditions.

**Table 2:** Linearity range, equation, correlation coefficient, the limit of detection, and quantification limit results for PGZ in rat plasma and tissues

Biological Samples	Linearity range (ng mL^−1^)	Equation^a^	Correlation coefficient (*R*^2^)	LOD (ng mL^−1^)	LLOQ (ng mL^−1^)
Plasma		*y* = 0.0068*x* + 0.5923	0.999		
Adipose tissue		*y* = 0.0019*x* + 0.0057	0.998		
Heart	1–500	*y* = 0.0018*x* + 0.0102	0.995	0.5	1.0
Kidney		*y* = 0.002*x* + 0.0107	0.997		
Brain		*y* = 0.0003*x* + 0.0028	0.992		
Bone		*y* = 0.0021*x* + 0.0043	0.998		

a *X* = concentration; *Y* = response factor.

**Table 3:** Percentage recovery, absolute matrix effect, intra, and inter-day accuracy and precision analysis of PGZ in rat plasma and tissues

Biological samples	Analyte concentration (ng mL^−1^)	Mean concentration found (ng mL^−1^)^a^	% recovery	Absolute matrix effect	Intra-day		Inter-day	
					Accuracy (%)	Precision (% RSD)	Accuracy (%)	Precision (% RSD)
Plasma	1	0.96 ± 0.07	96.00	0.93	95.89	8.12	93.29	9.87
	15	14.65 ± 1.12	97.67	0.94	98.05	7.95	95.27	8.09
	90	88.70 ± 5.66	98.56	0.97	98.55	6.67	97.68	8.11
	450	445.25 ± 25.75	98.94	1.15	98.78	6.09	97.06	7.55
Adipose tissue	1	0.94 ± 0.06	94.00	0.94	93.71	6.49	91.99	8.09
	15	14.39 ± 0.98	95.93	0.96	95.80	7.05	96.08	7.65
	90	86.45 ± 4.56	96.06	0.99	97.46	5.74	95.78	6.69
	450	440.01 ± 20.74	97.78	1.12	98.34	5.24	97.65	6.07
Heart	1	0.90 ± 0.05	90.06	0.91	89.97	6.17	88.11	7.89
	15	13.57 ± 1.09	90.47	0.94	88.89	8.28	87.75	8.97
	90	81.60 ± 5.01	90.67	0.97	91.27	6.15	89.89	6.88
	450	420.21 ± 30.75	93.38	1.09	92.88	7.09	90.93	7.34
Kidney	1	0.95 ± 0.08	95.70	0.93	96.49	8.45	95.00	10.15
	15	14.38 ± 0.97	95.87	0.95	95.00	6.77	94.96	8.74
	90	87.01 ± 6.01	96.68	0.98	96.68	7.90	96.00	9.09
	450	435.87 ± 30.01	96.86	1.06	96.86	6.81	95.78	7.47
Brain	1	0.94 ± 0.08	94.50	0.90	93.49	8.51	91.77	10.14
	15	14.24 ± 1.05	94.93	0.92	95.19	7.29	93.78	9.75
	90	85.50 ± 5.24	95.00	0.97	94.79	6.64	95.00	7.69
	450	428.09 ± 30.00	95.13	1.10	95.61	7.00	94.91	7.97
Bone	1	0.89 ± 0.09	89.90	0.91	90.00	10.11	88.06	11.65
	15	13.58 ± 1.21	90.53	0.92	89.97	8.90	87.61	10.46
	90	81.72 ± 6.99	90.80	0.94	91.00	8.50	90.09	10.09
	450	406.33 ± 25.40	90.30	1.01	90.64	6.20	89.99	9.55

a The data represents mean ± SD, *n* = 6.

**Table 4:** The stability study results of PGZ in rat plasma and tissues

Biological samples	Analyte concentration (ng mL^−1^)	Freeze–thaw (3 cycles at −70 ± 2 °C)		Short term (25 °C for 24 h)		Stock solution (25 °C for 24 h)	
		Accuracy (%)	Precision (% RSD)	Accuracy (%)	Precision (% RSD)	Accuracy (%)	Precision (% RSD)
Plasma	1	96.00	7.14	94.46	10.97	98.30	5.61
	15	97.87	6.68	95.38	9.12	98.67	3.54
	90	98.44	6.04	96.22	8.92	98.94	3.86
	450	98.89	6.09	96.89	8.54	99.29	2.89
Adipose tissue	1	92.55	6.11	89.97	9.89	97.42	4.21
	15	93.90	6.05	95.45	9.75	97.88	3.74
	90	95.60	5.47	95.01	8.09	98.34	2.37
	450	97.21	5.04	96.59	7.87	98.69	1.97
Heart	1	88.05	6.85	86.87	9.78	97.80	3.91
	15	89.19	7.08	86.97	8.17	98.21	3.05
	90	90.37	6.87	88.87	7.35	98.83	2.69
	450	92.88	7.09	89.95	7.69	99.09	2.07
Kidney	1	94.00	7.25	94.39	10.29	95.61	6.91
	15	94.67	6.07	94.54	9.14	97.91	5.61
	90	94.96	5.95	95.30	8.98	98.49	5.35
	450	95.32	6.02	95.67	8.34	98.87	3.31
Brain	1	92.09	6.22	89.82	11.94	96.84	3.95
	15	93.54	5.69	90.36	10.05	97.67	2.82
	90	93.89	5.13	92.41	9.39	97.97	2.36
	450	94.65	5.49	92.96	8.47	98.66	1.47
Bone	1	88.04	6.20	86.26	10.64	90.36	5.99
	15	88.47	5.90	87.89	9.56	93.49	4.88
	90	89.32	5.45	89.09	9.39	95.66	4.19
	450	90.16	5.15	89.97	8.26	96.19	3.78

### 3.3 Pharmacokinetic and tissue distribution studies in rat

**Fig. 2:**
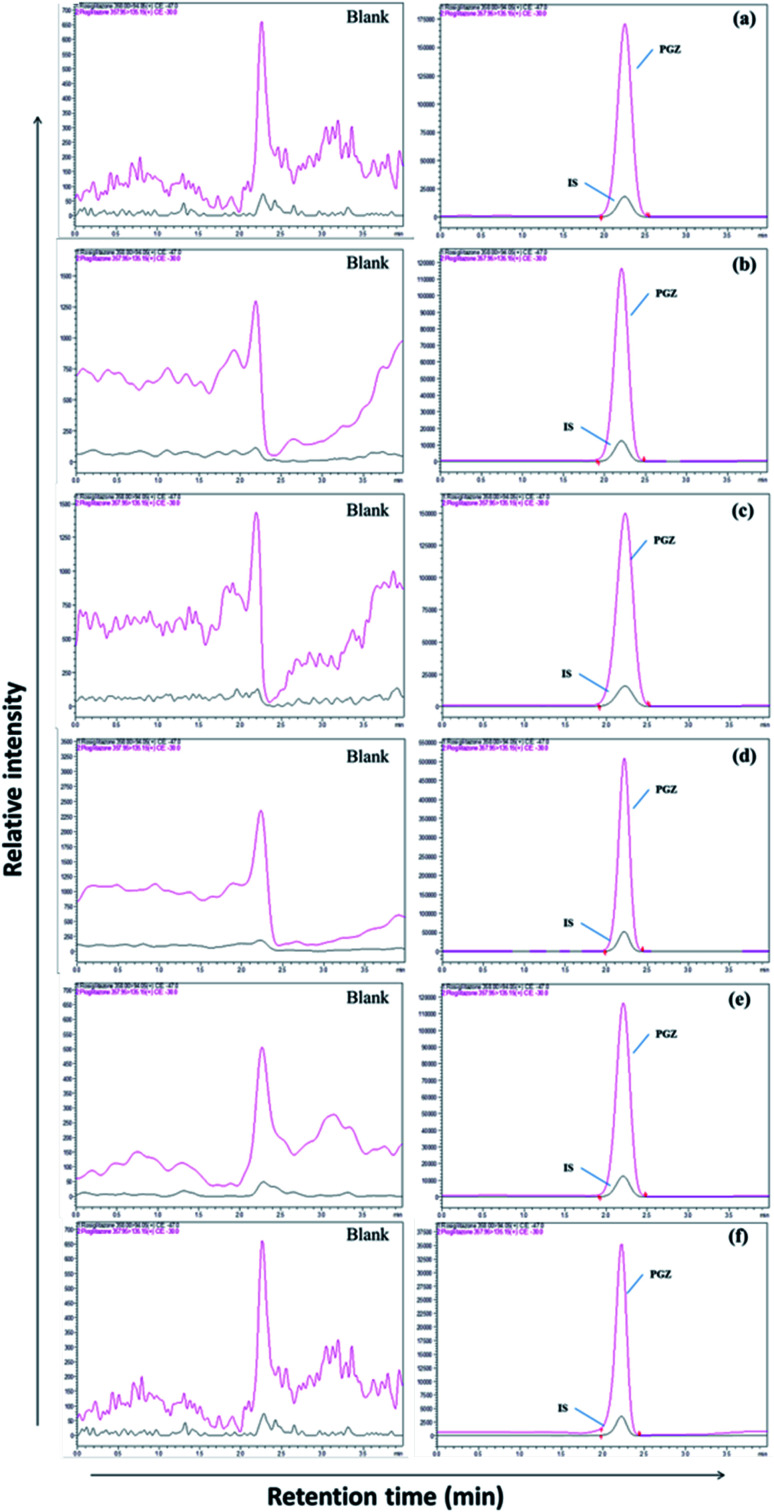
LC-MS chromatogram of PGZ (450 ng mL^−1^) and IS (RSZ 100 ng mL^−1^) in various biological matrices. (a) Plasma, (b) adipose tissue, (c) heart, (d) kidney, (e) brain, and (f) bone.

**Fig. 3:**
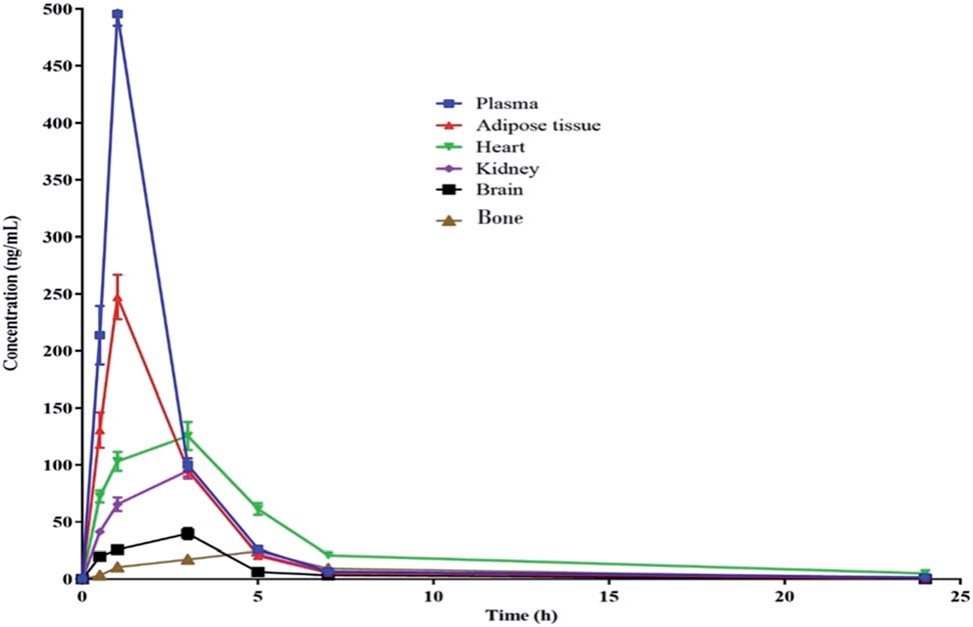
Pharmacokinetic profile of PGZ in various biological matrices after oral administration (10 mg kg^−1^) in rats.

The pharmacokinetic data of PGZ is given in Table 5, Fig. 2 and 3. The non-compartmental analysis was used to calculate the pharmacokinetics and tissue distribution of PGZ. The PGZ shows the highest *C*_max_ (in plasma), *T*_max_ (in bone), AUC_(0–24 h)_ (in plasma), AUC_(0–*α*)_ (in plasma), and *T*_1/2_ (in plasma) values of 495.03 ± 0.74 ng mL^−1^, 5.00 ± 0.49 h, 1056.58 ± 65.78 ng h^−1^ mL^−1^, 1069.38 ± 77.50 ng h^−1^ mL^−1^ and 5.62 ± 0.74 h, respectively, and the lowest *C*_max_ (in bone), *T*_max_ (in adipose), AUC_(0–24 h)_ (in brain), AUC_(0–*α*)_ (in brain), and *T*_1/2_ (in kidney) values of 24.50 ± 2.71 ng mL^−1^, 1.03 ± 0.04 h, 166.93 ± 15.70 ng h^−1^ mL^−1^, 167.12 ± 16.77 ng h^−1^ mL^−1^ and 2.78 ± 0.19 h, respectively. These pharmacokinetic variations indicate the non-specific tissue distribution of PGZ.

**Table 5:** Pharmacokinetic and tissue distribution of PGZ in the various biological matrices of rat

Parameters	Biological matrix^a^					
	Plasma	Adipose tissue	Heart	Kidney	Brain	Bone
C_max_ (ng mL^−1^)	495.03 ± 0.74	247.40 ± 18.75	125.60 ± 16.52	95.00 ± 9.87	39.66 ± 5.69	24.50 ± 2.71
*T*_max_ (h)	1.01 ± 0.05	1.03 ± 0.04	3.01 ± 0.35	3.10 ± 0.29	3.15 ± 0.24	5.00 ± 0.49
AUC_(0–24 h)_ (ng h^−1^ mL^−1^)	1056.58 ± 65.78	666.26 ± 35.49	780.52 ± 48.71	396.81 ± 33.19	166.93 ± 15.70	194.55 ± 21.29
AUC_(0–*∞*)_ (ng h^−1^ mL^−1^)	1069.38 ± 77.50	670.49 ± 36.74	818.20 ± 56.60	397.37 ± 30.05	167.12 ± 16.77	200.36 ± 26.80
*T*_1/2_ (h)	5.62 ± 0.74	4.38 ± 0.51	5.16 ± 0.66	2.78 ± 0.19	2.75 ± 0.31	4.37 ± 0.55

a The data represents mean ± SD, *n* = 3.

## 4 Discussion

Adipose tissue-specific delivery of PGZ using various delivery systems is an important strategy to overcome off-target side effects. In this regard, the development of a sensitive analytical method to accurately detect the PGZ concentrations in various tissue becomes essential for demonstrating the adipose tissue specificity of PGZ delivery systems. In the present study, we have developed and validated a sensitive LC-MS/MS method for measuring PGZ concentration in rat plasma and tissues. The mass conditions were optimized with the corresponding standard solution to obtain a high response of the analytes. ESI technique was adopted to determine their ionization modes with the mobile phase. PGZ and IS (RSZ) had a more efficient intense mass resolution in positive mode with a protonated molecular ion [M + H]^+^ of *m*/*z* 357.95 for PGZ and 358.00 for IS (Fig. 1). Moreover, the behavior of the analyte and the sensitivity of detection was also dependent on the CE. The CE of −30 eV and −47 eV was optimal for PGZ and IS, respectively. The precursor → product ion transitions (MRM) of PGZ and IS was *m*/*z* 357.95 → 135.15 and *m*/*z* 358.00 → 94.05, respectively, which were in agreement with the previous reports.^26–30^

The separation of PGZ and IS was performed on a YMC Pro C_18_ column (100 mm × 4.6 mm, 3μ) with the mobile phase consists of 0.1% formic acid and acetonitrile (5 : 95, v/v) at the flow rate of 0.7 mL min^−1^. The retention time of PGZ and IS were found to be 2.45 and 2.46 min, respectively, with a total run time of 4.00 min (Fig. 2). The optimized chromatographic condition provided a high peak area compared to previously developed methods, and the analytes were unaffected by endogenous interferences from rat plasma/tissue homogenate. From the literature, few LC-MS/MS reported methods were previously developed and reported for the determination of PGZ in plasma of rat/human/dog.^23,26–28,31–35^ The reported methods have their individual advantages such as short run time, simple sample preparation technique, requiring low plasma volume and with improved method sensitivity. Further, the previously reported methods were developed for the simultaneous estimation of the pharmacokinetics of PGZ along with other drugs in conventional dosage forms such as tablets. None of these methods, however, can be adapted for the validation of adipose tissue-specific delivery systems of PGZ by measuring its concentration in the target (adipose tissue) and off-target tissues (bone, brain, heart, and kidney). Table SIII† summarizes and compares the various reported methods for the estimation of PGZ in biological matrices. In this study, we have successfully developed an LC-MS/MS method with short runtime (4.0 min), simple sample preparation technique requiring small sample volume (200 μL) with recoveries ranging from 89.00 to 98.94%. Further, the developed method was highly sensitive with LOD and LLOQ of 0.5 ng mL^−1^ and 1.0 ng mL^−1^, respectively for accurate determination of PGZ in plasma and tissues so that the method can be used for validating adipose tissue-specific delivery systems of PGZ in addition to conventional dosage forms.

## 5 Conclusion

A rapid and sensitive LC-MS/MS method has been developed and validated for the determination of PGZ in different biological matrices. Compared with the previous methods, the present method has significant advantages and can estimate PGZ in various tissues. The present method can be adopted for validating adipose tissue specific delivery systems of PGZ such as solid lipid nanoparticles, dendrimers, carbon nanomaterials, metallic nanoparticles, *etc.*, in addition to the conventional dosage form of PGZ.

## Abbreviations

T2DM: Type-2 diabetes mellitus
PGZ: Pioglitazone
RGZ: Rosiglitazone
LC-MS/MS: Liquid chromatography-tandem mass spectrometry
IS: Internal standard
HQC: High quality control
MQC: Medium quality control
LQC: Low-quality control
LLQC: Lower limit quality control
ESI: Electro-spray ionization
MRM: Multiple reaction monitoring
IAEC: Institutional animal ethics committee

## Funding

The present work was funded by DST-Nanomission (DST/NM/NB/2018/227 (G), and (C)), Govt. of India, New Delhi.

## Author contributions

Ms Kusuma Kumari conceptualized, analyzed, and majorly contributed in writing manuscript. Dr Praveen supervised, proof-read the manuscript. Mr Ravi Kiran contributed in research work and proof-read. Dr Narenderan helped in analytical work and interpretation of the data. Dr Vishwanath, Dr Babu & Dr Krishnaveni provided valuable insights and proof-read the manuscript.

## Conflicts of interest

The authors reported no potential conflict of interest.

## Supplementary Material

RA-011-D1RA01126J-s001
