# Supplementary material for: Development and validation of a sensitive LC-MS/MS method for pioglitazone: application towards pharmacokinetic and tissue distribution study in rats
Source: RSC Adv. 2021 Mar 18;11(19):11437–43. doi: 10.1039/d1ra01126j (PMC8695949; doi:10.1039/d1ra01126j)
Supplement: RA-011-D1RA01126J-s001 [file RA-011-D1RA01126J-s001.pdf]

Electronic Supplementary Material (ESI) for RSC Advances.
This journal is © The Royal Society of Chemistry 2021

Development and validation of a sensitive LC-MS/MS method for Pioglitazone: Application towards pharmacokinetic and tissue distribution study in rats

Kusuma Kumari G[a]; Praveen Thaggikuppe Krishnamurthy*; Ammu V. V. V. Ravi Kiran[a]; Vishwanath Kurawattimath[b]; Narenderan S.T[c]; Babu B[c]; Krishnaveni Nagappan[c]

[a] Department of Pharmacology, JSS College of Pharmacy, JSS Academy of Higher Education & Research, Ooty, Nilgiris, Tamil Nadu- 643001, India.

[b] Drug Metabolism and Pharmacokinetics-Toxicology Division, Sai Advantium Pharma Ltd., Pune-411 057, India

[c] Department of Pharmaceutical Analysis, JSS College of Pharmacy, JSS Academy of Higher Education & Research, Ooty, Nilgiris, Tamil Nadu- 643001, India.

*Corresponding author: Dr. Praveen Thaggikuppe Krishnamurthy
Professor and Head
Department of Pharmacology
JSS College of Pharmacy, Ooty
e-mail: praveentk7812@gmail.com, praveentk@jssuni.edu.in

Table S-I: A staggering collection of blood and tissue samples for pharmacokinetic studies of PGZ in rats

Animals	0 hrs	0.5hrs	1hrs	3 hrs	5hrs	7 hrs	24 hrs
1	B	B	NC	B	NC	B	B
2	B	B	NC	B	NC	B	T
3	B	B	NC	B	NC	B	T
4	B	B	NC	B	NC	T	X
5	B	B	NC	T	NC	X	X
6	B	T	X	X	X	X	X
7	B	NC	B	NC	B	B	B
8	B	NC	B	NC	B	B	T
9	B	NC	B	NC	B	B	T
10	B	NC	B	NC	B	T	X
11	B	NC	B	NC	T	X	X
12	B	NC	T	X	X	X	X

B: blood**; T:** tissues; **NC**: no collection; X: None

Table S-II: Mobile phase compositions used for LC-MS/MS method development

S.No	Organic phase		Aqueous phase	Aqueous: organic Ratio	Inference
1.	Ammonium acetate	1mM<br>2mM<br>3mM<br>4mM<br>5mM	Acetonitrile	50:50<br>60:40<br>70:30<br>80:20<br>90:10<br>95:05	Peak tailing and Spilt peak
2.	Ammonium formate	1mM<br>2mM<br>3mM<br>4mM<br>5mM	Acetonitrile	50:50<br>60:40<br>70:30<br>80:20<br>90:10<br>95:05	Peak tailing and fronting
3.	Acetic acid	0.1% v/v<br>0.5% v/v<br>1% v/v<br>2% v/v<br>3% v/v	Acetonitrile	50:50<br>60:40<br>70:30<br>80:20<br>90:10<br>95:05	Spilt peak and Peak tailing
4.	Formic acid	0.1% v/v	Acetonitrile	50:50	Fronting
				60:40	Peak tailing
				70:30	Peak tailing
				80:20	Peak tailing
				90:10	Peak tailing
				95:05	Good peak

Table S-III: Comparison of various methods available for the estimation of Pioglitazone in biological matrices

Authors	Species and Biological matrix	Pharmacokinetics data				Various method	Method validation					Collision energy (eV)	Run time (min)	Retention Time (Min)	Ref.
		C_{max} (ng/ml)	$t_{1/2(hr)}$	T_{max} (hr)	AUC (0-24 hrs) ng/h/ml		Linearity range (ng/ml)	Precision (Intra & Inter day) %	Accuracy (Intra &inter day) %	LLOQ ng/ml	LOD ng/ml				
Gananadhamu et al.	RP	6062	4.9	1.5	55116	LC-ESI-MS/MS (SM-Sitagliptin)	8 - 1571	9.5-9.8 & 6.1-6.5	101.0-102.8 & 96.3-98.2	8.2	-	39	5	2.81	(1)
Elgawish et al.	RP	1920	10.8	1.0	18214	LC-MS/MS (SM-metformin)	1 - 2500	0.6-14.3 & 0.6-10.3	95.8–104.6 & 93.2-95.5	14.7	0.95	40	15	14.72	(2)
Sengupta et al.	RP	3870	-	3.6	4158	LC-MS/MS (SM-Telmisartan)	5–10000	3.1 -6.7 & 5.6 -8.1	94.2-106.1 & 92.3-98.9	5.00	-	40	-	-	(3)
Abdel-Ghany et al.	HP	1462	5.9	1.5	12178	LC-MS/MS (SM-Alogliptin)	25-2000	2.5 – 1.4 & 1.9 –0.5	99.4-100.4 & 94.6-97.4	25.0	-	40	-	-	(4)
Hess et al.	HP	-	-	-	-	LC-MS (SM-Hydroxy tolbutamide, Vildagliptin)	1-750	5.4 -10.1 & 8.9-13.0	96.4-100.5 & 94.9-98.6	25.0	-	43	-	-	(5)
Jagadeesh et al.	HP	709	-	4.5	20640	LC-MS (SM-Metformin)	15–2500	13.2-15.1 & 41.8-42.7	88.0-97.4 & 84.3-89.5	15.0	-	55	4	2.6	(6)
Zhang et al.	DP	2,640	5.8	0.9	7121.9	LC-MS/MS (SM-Metformin)	1-1000	9.6 & 6.5 & 9.7-6.8	90.7-101.6 & 89.6-94.3	1.0	0.50	31	8	6.1	(7)
Sengupta et al.	HP	-	-	-	-	LC-MS/MS (SM)-Glimepride	2–1000	2.5-2.7& 2.6-2.7	96.0–105.1 & 94.3-97.0	2.5	-	60	6	2.71	(3)
Khadiga et al.	HP	1847	8.8	3.7	26572	LC-MS/MS (SM-Aloglitpin)	10-3000	10-10.6 & 9.6-10	96.6-103.3 & 90.2-97.6	10	-	60	-	3.01	(8)
Current method	RP	495	5.6	1.0	1056	LC-MS/MS	1-500	6.0-8.1 & 7.5-9.8	93.2-97.6 & 95.8-98.7	1.0	0.50	30	4	2.45	--
	RA	247	4.38	1.0	666			5.2-7.0 & 6.0-8.0	93.7-98.3 & 91.9-						

									97.6						
	RH	125	5.16	3.0	780			6.1-8.2 & 6.8-8.9	88.8-92.8 & 87.7-90.9						
	RB	24.	4.37	5.0	194			6.2-10.1 & 9.5-11.6	89.9-91.0 & 87.6-90.0						
	RK	95	2.78	3.1	3969			6.7-8.4 &-7.4-10.1	95.0-96.8 & 94.9-96.0						
	RBr	39	2.75	3.1	166			6.64-8.51%&7.69-10.14%	93.4-95.6 & 91.7-95.0						

AUC – Area Under curve; LLOQ – Lower limit of Quantification; ESI- Electron Spray Ionization, SM- Simultaneous method, RP- Rat plasma, RA- Rat adipose tissue, RH- Rat heart, RB-Rat bone, RK- Rat kidney, RBr- Rat brain, HP- Human plasma, DP- Dog plasma.

1. Gananadhamu S, Laxmikanth V, Shantikumar S, Sridhar V, Geetha C, Sandhya C. Rapid LC-ESI-MS-MS Method for the Simultaneous Determination of Sitagliptin and Pioglitazone in Rat Plasma and Its Application to Pharmacokinetic Study. American Journal of Analytical Chemistry. 2012;03(12):849-58.
2. Elgawish MS, Nasser S, Salama I, Abbas AM, Mostafa SM. Liquid chromatography tandem mass spectrometry for the simultaneous determination of metformin and pioglitazone in rat plasma: Application to pharmacokinetic and drug-drug interaction studies. Journal of Chromatography B. 2019;1124:47-57.
3. Sengupta P, Chatterjee B, Mandal UK, Gorain B, Pal TK. Development and validation of a high throughput LC–MS/MS method for simultaneous quantitation of pioglitazone and telmisartan in rat plasma and its application to a pharmacokinetic study. Journal of Pharmaceutical Analysis. 2017;7(6):381-7.
4. Abdel-Ghany MF, Ayad MF, Tadros MM. Enhanced LC–MS/MS analysis of alogliptin and pioglitazone in human plasma: Applied to a preliminary pharmacokinetic study. Journal of Chromatography B. 2017;1058:93-101.
5. Hess C, Musshoff F, Madea B. Simultaneous identification and validated quantification of 11 oral hypoglycaemic drugs in plasma by electrospray ionisation liquid chromatography–mass spectrometry. Analytical and Bioanalytical Chemistry. 2011;400(1):33-41.
6. Jagadeesh B, Bharathi DV, Pankaj C, Narayana VS, Venkateswarulu V. Development and validation of highly selective and robust method for simultaneous estimation of pioglitazone, hydroxypioglitazone and metformin in human plasma by LC–MS/MS: Application to a pharmacokinetic study. Journal of Chromatography B. 2013;930:136-45.
7. Zhang X, Peng Y, Wan P, Yin L, Wang G, Sun J. Simultaneous Determination and Pharmacokinetic Study of Metformin and Pioglitazone in Dog Plasma by LC-MS-MS. Journal of Chromatographic Science. 2013;52(1):52-8.
8. Kelani KM, Rezk MR, Badran OM, Elghobashy MR. Determination of pioglitazone, its metabolite and alogliptin in human plasma by a novel LC-MS/MS method; application to a pharmacokinetic study. Journal of Chromatography B: Analytical Technologies in the Biomedical and Life Sciences. 2019;1132.
